# Association between Handgrip Strength and the Systemic Immune-Inflammation Index: A Nationwide Study, NHANES 2011–2014

**DOI:** 10.3390/ijerph192013616

**Published:** 2022-10-20

**Authors:** Dongzhe Wu, Xiaolin Gao, Yongjin Shi, Hao Wang, Wendi Wang, Yanbin Li, Zicheng Zheng

**Affiliations:** 1 Sports Rehabilitation Center, China Institute of Sport Science, Beijing 100061, China; 2 Department of Physical Education and Art, China Agricultural University, Beijing 100083, China; 3 Human Health Science Research Department, Tokyo Metropolitan University, Tokyo 116-8551, Japan; 4 Human and Social Sciences, Chemnitz University of Technology, 09126 Chemnitz, Germany

**Keywords:** systemic immune-inflammatory index, handgrip strength, cross-sectional study

## Abstract

(1) Background: The Systemic immune-inflammatory index (SII) has been proven to be an effective biomarker of human immune and inflammatory levels and has prognostic significance for most diseases. Handgrip strength (HGS) is a simple and low-cost strength measurement method, which is not only highly correlated with overall muscle strength but also accurately and reliably predicts the risk of multiple chronic diseases and mortality; (2) Purpose: Association between HGS and the SII is unclear. The purpose of this study was to investigate the association between HGS and the SII in American adults; (3) Methods: We used the data from the 2011–2012 and 2013–2014 cycles of the National Health and Nutrition Examination Survey (NHANES), involving a total of 8232 American adults (aged 18–80 years). The SII was calculated as the Platelet count × Neutrophil count/Lymphocyte count; HGS was recorded as the ratio of the sum of the highest grip-strength values of each hand to body mass index taken as the relative grip strength. A weighted generalized linear regression model and analysis of restricted cubic spline regression, adjusted for confounding factors, were used in this study to assess associations between HGS and the SII in American adults; (4) Results: There was a negative correlation between the HGS and the SII of different sexes (*p* < 0.05), and there was a significant negative nonlinear relationship between the HGS and the SII in males (*p* for nonlinear = 0.0035), and the SII showed a downward trend with the increase in the HGS in males (Q2: β = −61.03, *p* = 0.01; Q3: β = −61.28, *p* = 0.04, Q4: β = −64.36, *p* = 0.03, *p* for trend = 0.04), when the HGS exceeds 3.16, with the HGS increasing, the downward trend of increasing the SII slowed down. The nonlinear relationship between the HGS and the SII in females was not significant (*p* for nonlinear = 0.1011), and the SII showed a linear downward trend with the increase in the HGS (Q2: β = −24.91, *p* = 0.25; Q3: β = −62.01, *p* = 0.03, Q4: β = −74.94, *p* = 0.03, *p* for trend = 0.01); (5) Conclusions: HGS is inversely and independently associated with SII levels, and although the limited cubic spline regression analysis showed gender differences, the overall trend of the HGS and the SII in different genders was consistent, with both showing that the SII decreased with increasing the HGS. In addition, HGS has high general applicability based on its ease of measurement; it is possible to understand one’s own grip-strength level through routine grip-strength tests, and to make preliminary predictions on the current level of immunity and inflammation in the body.

## 1. Introduction

Many studies have confirmed that the level of inflammation and immune function can directly affect the health of the human body, and chronic inflammation and decreased immune function directly or indirectly induce most chronic diseases (such as cancer, diabetes, Parkinson’s disease, and osteoporosis) [1,2,3,4,5,6,7,8]. Therefore, the regulation of inflammation levels and immune function may be of great significance for the prevention and treatment of chronic diseases and improvement of national health.

The systemic immune-inflammatory index (SII) is considered a stable and accurate indicator of the overall or local immune response and inflammation level of the human body [9,10,11,12,13]. This indicator is calculated using three inflammatory factors: platelets, neutrophils, and lymphocytes. Relevant studies have confirmed that the SII exhibits strong prognostic value for tumors and chronic diseases induced by chronic inflammation or immune dysfunction [9,10,11,12]. In addition, previous studies have found that the SII has a higher prognostic accuracy than other traditional inflammatory factors (such as C-reactive protein (CRP), serum albumin, and triglyceride levels) in patients with severe pancreatitis [13].

Skeletal muscle is the most abundant tissue in the human body and plays an important role in maintaining a healthy environment owing to its high degree of remodeling. Muscle mass changes dynamically in real-time with factors such as physical activity, disease, and aging [14]. According to the latest epidemiological surveys, the prevalence of sarcopenia caused by population aging is increasing every year, and the decline in muscle mass leads to serious disability and mortality [15,16]. Handgrip strength (HGS) is a simple and low-cost strength measurement method, which is not only highly correlated with overall muscle strength but also accurately and reliably predicts the risk of multiple chronic diseases and mortality [7,17,18,19,20,21,22]. A cross-sectional study of the Korean National Health and Nutrition Examination Survey (KNHANES VI) confirmed that lower HGS was associated with an increased risk of depression in Korean adults (young, middle-aged, and older), suggesting that increasing muscle strength could prevent depression in Korean adults [23]. A recent cross-sectional study of the NHANES confirmed that HGS in older adults was significantly associated with better cognitive performance, both globally and in a variety of domains, such as memory, language, attention, and subjective cognition [24]. A large population study by Soto et al. found that HGS was associated with morbidity and mortality in endometrial, gallbladder, colorectal, liver, all-cause, and breast and kidney cancers, independent of major confounders (comorbidities, diet, and physical activity), highlighting HGS as an independent risk predictor for several cancer sites [25]. An Urban Rural Epidemiology China Study by Liu et al. found that low HGS in hypertensive patients is associated with the risk of major CVD incidence, CVD mortality, and all-cause mortality, and high HGS levels appear to reduce the long-term risk of death in hypertensive patients [26]. Christina et al. concluded that physical fitness, especially muscle strength (grip strength), may increase health-related quality of life and is an important source of health in older age, suggesting that grip strength can be used as an indicator of health-related quality of life [27]. Recently, an up-to-date and comprehensive review of “Handgrip strength and Health” suggested HGS as a prognostic “biomarker” for specific outcomes across the lifespan of human beings, among both current and future populations [28].

The SII and HGS are both known to provide additional valuable information for risk prediction for many chronic diseases and mortality, but the relationship between the SII and HGS is currently unclear. This study aimed to determine the association between HGS and the SII among American adults. Based on previous studies, we propose there is an association between HGS and the SII, and that the SII may decrease with the increase in HGS.

## 2. Materials and Methods

### 2.1. Study Design

The subjects of this study were drawn primarily from the National Health and Nutrition Examination Survey (NHANES), a nationally representative survey led by the Centers for Disease Control and Prevention, to assess the health and nutritional status of American adults and children. The Centers for Disease Control and Prevention conducted surveys and published data on a 2-year cycle. The survey utilized a multistage probability sampling design to examine a nationally representative sample of approximately 10,000 non-institutionalized individuals across the United States.

During home interviews, participants were asked questions about demographic, socioeconomic, diet, and health-related parameters, and underwent a physical examination that included medical, dental, and physiological measurements, among others. A total of 19,931 individuals were sampled by the NHANES between 2011 and 2014. Our study inclusion criteria were as follows: (i) adults over 18 years old; (ii) fully participated in the handgrip-strength test; (iii) participated in the blood test. After excluding those who did not meet the criteria or had missing data (11,699 people), 8232 people were included as the participants in the present study. The study was approved by the Research Ethics Review Board of the National Center for Health Statistics, and participants signed an informed consent form.

### 2.2. Procedures

#### 2.2.1. Blood Extraction

Blood tests (complete blood count, CBC) of the NHANES 2011–2014 were analyzed on a Coulter HMX (Coulter Electronics Ltd., Bedfordshire, UK) in 2011–2012 and the Beckman Coulter DXH 800 (Beckman Coulter, Brea, CA, USA) in 2013–2014 using mixed EDTA blood samples from participants. 

Neutrophil, lymphocyte, and platelet counts were collected for analysis. The systemic immune-inflammation index (SII) was calculated from the CBC values using the following formula: *platelet count × neutrophil count* *÷ lymphocyte count* [11].

#### 2.2.2. Handgrip Strength

Handgrip strength (HGS) was measured by Takei dynamometer (TKK 5401; Takei Scientific Instruments, Tokyo, Japan). The participants were asked to maintain an upright posture, with their arms vertically downward, and hold the dynamometer for strength testing. The test was repeated three times for both hands (dominant hand and non-dominant hand), with a 60-s interval between each measurement, and the highest grip-strength value of each hand was taken. The ratio of the sum of the highest grip-strength values of each hand to BMI was taken as the relative grip strength [29,30,31,32]. 

#### 2.2.3. Covariates

Covariates that may have influenced the results were included in this study: sex (male, female), age (<20, 20–29, 30–39, 40–49, 50–59, ≥60), education level (below high school level, high school, above high school), race (black, white, Mexican, other races), poverty-to-income ratio (PIR, calculated by dividing family (or individual) income by the poverty guidelines specific to the survey year; <1.3, 1.3–3.49, ≥3.5), BMI (<25, 25–29.9, ≥30), smoking status (never smoked, former smoker, current smoker), alcohol status (never, former, mild, moderate, heavy), hypertension (yes, no), diabetes (yes, no), hyperlipidemia (yes, no).

### 2.3. Statistical Analysis

R Studio (4.2.0, US) was used for statistical analysis. During the data analysis process, the sampling weight was used to consider the complex survey design (for example, the selection probability was not equal), and comprehensive weight analysis and weighting processing were performed on the final valid data. 

Continuous variables were presented as weighted means and standard errors, and categorical variables were presented as weighted percentages. The chi-square and *t*-tests were used for categorical variables and continuous variables, respectively, to compare the distribution and mean differences of correlated variables among individuals of different sexes. A weighted generalized linear regression model was used to analyze the association between HGS and the SII for complex survey samples, and covariates were controlled to explore the linear relationship between HGS and the SII. Weighted generalized linear models were adjusted for covariates and further stratified by sex. A restricted cubic spline plot (RCS) was used to detect potential nonlinear relationships between HGS and the SII, and the RCS models were adjusted for covariates and further stratified by sex. All statistical tests were two-sided, and a *p*-value < 0.05 was statistically significant.

## 3. Results

### 3.1. Baseline Characteristics of Participants

A total of 8232 participants were included in this study, including 4100 women (49.8%) and 4132 men (50.2%). Table 1 shows the distribution of participant characteristics for each quartile of the SII. HGS, sex, age, race, BMI, education, smoking status, alcohol status, diabetes, hypertension, and hyperlipidemia were all significantly different between the SII quartile groups (*p* < 0.05). 

In contrast, people over 60 years of age, women, white ethnicity, higher BMI, and highly educated people were associated with higher SII levels. Non-drinkers had lower SII levels, but this trend was not significant in non-smokers. In the population with comorbidities, higher SII levels were seen in patients with hyperlipidemia, but not in those with diabetes and hypertension. We observed that HGS was highest in the lowest SII quantile and showed a downward trend with increasing the SII.

### 3.2. The Association between the SII and Handgrip Strength

Table 2 shows the relationship between HGS and the SII in the three weighted generalized linear regression models. In the fully adjusted model (Model III), the highest quartile of HGS was more negatively associated with the SII than the lowest quartile of HGS (β = −77.31, −129.58~−25.04, *p* = 0.01), and remained relatively stable in different models. Compared with the lowest quartile of HGS, the second and third quartiles of HGS were significantly negatively correlated with the SII, and the above correlations were all statistically significant (Q2: β = −35.75, −66.93~−4.56, *p* = 0.03; Q3 = −79.49, −115.44~−43.55, *p* < 0.001). The same trend was observed in the sensitivity analysis (*p* for trend < 0.01).

In the subgroup analysis stratified by sex, male HGS and the SII remained relatively stable across different models and, in the fully adjusted model (Model III), HGS and the SII were associated (Q2: β = −61.03, −101.14~−20.92, *p* = 0.01; Q3: β = −61.28, −116.71~−5.86, *p* < 0.05; Q4: β = −64.36, −118.25~−10.46, *p* < 0.05). Sensitivity analysis showed the same trend (*p* for trend < 0.01).

Compared with the lowest quantile of female HGS, females’ HGS third and fourth quartiles and the SII remained relatively stable across the different models. In the fully adjusted model (Model III), the correlation between HGS and the SII was significant (Q2: β = −24.91, −101.14~−20.92, *p* = 0.25; Q3: β = −62.01, −117.07~−6.94, *p* < 0.05; Q3: β = −74.94, −137.21~−12.66, *p* < 0.05). Sensitivity analysis showed the same trend (*p* for trend < 0.05).

### 3.3. Analysis of Restricted Cubic Spline Regression

**Figure 1:**
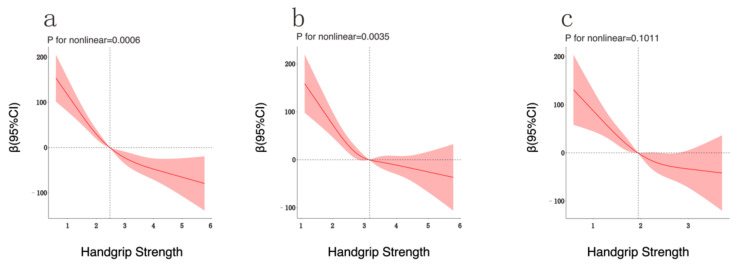
Analysis of Restricted Cubic Spline Regression. Legend: Adjusted restricted cubic spline models ((**a**): overall, (**b**): male, (**c**): female) showed the association between HGS and SII in all participants. Model adjusted for sex, age, race, BMI, education, PIR, smoking, alcohol, diabetes, hypertension, and hyperlipidemia. Solid and long dashed lines represent the estimated regression coefficient Beta and its 95% confidence interval.

In restricted cubic spline regression, adjusting for different covariates, we found a significant nonlinear relationship between HGS and the SII (*p* = 0.0006, Figure 1a). Figure 1a shows a decreasing trend of the SII with increasing the HGS. When the HGS exceeds 2.48, the decreasing trend of the SII becomes slower with increasing the HGS. In the subgroup analysis of different sexes, it was found that there was a significant nonlinear relationship between male HGS and the SII (*p* = 0.0035, Figure 1b), while female HGS had no significant nonlinear relationship with the SII (*p* = 0.1011, Figure 1c). Figure 1b shows that the SII decreased with increasing the HGS in males. When the HGS exceeded 3.16, the decreasing trend of the SII slowed down with increasing the HGS. Although the nonlinear analysis results of the three restricted cubic splines were slightly different, the overall changing trends of the dependent and independent variables in each graph were relatively consistent.

## 4. Discussion

In this cross-sectional study of 8232 participants, we investigated the association between HGS and the SII in American adults. The main purpose of this study was to explore the potential relationship between HGS and the SII, and a negative correlation between HGS and the SII was confirmed in this study. Our findings reinforce current public health physical activity guidelines (emphasizing the importance of engaging in muscle-strengthening activities) [33], suggesting that higher levels of HGS may be associated with immune function and levels of inflammation in human beings.

Absolute values of HGS are closely related to body weight, and the use of it as an indicator of muscle strength, without correcting for body weight, may be an important reason for the contradictory results of many studies [29,30,32,34]. Several studies have shown that relative values of HGS are better than absolute grip strength in predicting some chronic diseases [18,29,30], so this study chose relative grip strength as the main independent variable to explore its potential association with the SII. A study by Jun Kim et al. confirmed that higher serum hypersensitive C-reactive protein (hs-CRP) in older men was associated with lower HGS [31], further suggesting that chronic low-grade systemic inflammation may, alongside aging, be a common risk factor for simultaneous deterioration of muscle and bone. The association analysis of HGS and hs-CRP in postmenopausal women conducted by Son et al. supplemented the above studies, which found that serum hs-CRP in postmenopausal women was negatively correlated with HGS [29]. A study on the association analysis of HGS and inflammatory biomarker profiles in the elderly confirmed that inflammatory signatures such as hs-CRP and albumin were independently associated with baseline HGS, and future studies linking more inflammatory signatures to muscle strength are needed to confirm these findings in older adults [28]. Brinkley et al. found a high correlation between inflammatory biomarkers (CRP and IL-6) and HGS [35]. The study by Schaap et al. further confirmed that TNF-α and its soluble receptor (IL-6sR) exhibited the most consistent associations with decreases in muscle mass and strength, finding that HGS was highly inversely correlated with TNF-α and IL-6sR [36]. The above evidence provides further support for the development and implementation of this study. Obviously, the association between HGS and inflammation has been confirmed, but there are still few studies on the association analysis of HGS and immune function. Based on the effective application of the SII in epidemiological studies in the past, this study adopted the SII, and it is reasonable to assess the association of muscle strength with levels of inflammation and immune function.

The results of this study are consistent with those of previous related studies [15,29,31,36,37] and, while proving that higher HGS is highly correlated with lower SII, we analyzed the nonlinear relationship between HGS and the SII using restricted cubic spline regression analysis to further explain the association. The physiological mechanisms related to the association between HGS, immune function, and inflammation are relatively complex. This study attempted to explore these physiological mechanisms by logically sorting out the limited number of previous studies.

Immune dysfunction and chronic inflammation are combined risk factors for sarcopenia and decreased muscle strength [5,15,16,29,36,38,39,40,41]. It is known that HGS is an important diagnostic indicator of sarcopenia, and this indicator is highly correlated with overall muscle strength. Some studies have suggested that skeletal muscle can directly regulate immune function and the degree of inflammation in the human body [16,42]. Therefore, we believe that the relationship between muscle strength, chronic inflammation, and immune function is not unidirectional. The perception of skeletal muscle as a mere motor organ has changed over the past two decades, and muscle has become recognized as a specialized organ with immunomodulatory properties [7].

Interleukin-6 (IL-6) is a well-known myokine, has two-way pro-inflammatory/anti-inflammatory effects. After moderate exercise, muscles release a large amount of IL-6 into the circulating blood, where it acts as an anti-inflammatory factor by regulating satellite cell function and enhancing glucose metabolism, thereby contributing to the recovery of skeletal muscle and improvement of muscle strength [14,43,44,45,46]. However, long-term exposure to, and high concentrations of IL-6 are accompanied by a large degree of activation of pro-inflammatory factors such as TNF-α, thereby weakening the ability of muscle anabolism and energy homeostasis to induce muscle atrophy and muscle strength decline [29,40,43].

Muscle strength depends, to a certain extent, on the regenerative potential of skeletal muscle after exercise, and the regenerative potential of skeletal muscle is mainly affected by the interaction between skeletal muscle and immune cells [47,48]. In response to post-exercise muscle injury, immune cell infiltration of skeletal muscle promotes muscle recovery and strengthening by removing apoptotic and necrotic cells and secreting several growth factors required for satellite cell proliferation and differentiation.

The immune system plays an important role in the maintenance and support of skeletal muscle, and studies have suggested that levels of immune function may affect muscle growth [7,36,37,46,47,48,49,50]. Age-related immune dysregulation and aging may be important reasons for the worsening of sarcopenia, and grip strength, an important indicator for assessing muscle strength in sarcopenia, may be potentially associated with immune function to a certain extent [5,15,16,36,39,40,41,42,48]. As people age, the immune system undergoes immunosenescence. Aging leads to increased levels of pro-inflammatory factors (especially CRP, TNF-α, and IL-6) in the blood, thereby inducing long-term chronic inflammation and decreased immune function, which is also a major factor for the increased chronic disease risk in the elderly [38,39]. Relevant studies have shown that Interleukin-15 (IL-15) plays an important role in immune function during human aging. It not only acts on the proliferation, activation, and distribution of B-cells and natural killer cells but also affects the maintenance of T-cell activity [49,50,51,52]. The contraction of skeletal muscle during exercise releases a large amount of IL-15 into the circulating blood to quickly improve immune function [41]. Notably, however, skeletal muscle production and release of IL-15 are affected by adenosine 5′-monophosphate-activated protein kinase (AMPK) levels, and AMPK activity declines with increasing age [53,54]. Therefore, it is unclear whether muscle strength can counteract the negative effects of aging on IL-15 production and release. Recently, a study by Lopez et al. showed that high levels of HGS were associated with lower total white blood cell count, suggesting that healthy muscle levels could help boost immune system function in adolescents [33]. The above evidence provides valuable theoretical reference for the results of this study. Although there is less research to prove the relationship between HGS and immune function, based on the above limited evidence, HGS is an important indicator of human muscle strength. The decline of immune function induces acute and chronic inflammation in the whole body and mediates and affects the repair of skeletal muscle and regeneration, which in turn triggers a decline in HGS.

Our study had several advantages. This is the first study to explore the relationship between HGS, degree of inflammation, and immune function based on the SII indicators. This study used a large representative sample from the American NHANES. We abandoned the previous methods of using absolute one-handed (or dominant hand) HGS and instead used the relative HGS value, which is currently considered to be the most accurate, as the main research index of the study. While studying the linear relationship between the indicators, restricted cubic spline regression analysis, commonly used in epidemiology, was included to further explore the potential correlation between the indicators. However, our study had some limitations. Some classic inflammatory factors (TNF-α, IL-6, IL-10, etc.) have not been recorded by NHANES, so relevant indicators cannot be included in this study for analysis to obtain more comprehensive results. In addition, although HGS is a typical indicator for evaluating muscle strength, if experimental conditions allow, more muscle strength (lower limb muscle strength) and muscle mass evaluation indicators should be included as much as possible for joint explanation. Because this study is based on the NHANES large population survey, it cannot provide a more comprehensive assessment of muscle strength, so this is a limitation in our study. This study provides scientific data reference for future research. We will also include strength testing of different muscle groups and assessment of skeletal muscle mass in future research to further explore the relationship between muscle strength and immunity and inflammation.

## 5. Conclusions

In conclusion, data were collected from a nationally representative survey that HGS is inversely and independently associated with SII levels, and although the limited cubic spline regression analysis showed gender differences, the overall trend of HGS and the SII in different genders was consistent, with both showing that the SII decreased with increasing the HGS.

In addition, HGS has high general applicability based on its ease of measurement; it is possible to understand one’s own grip-strength level through routine grip-strength tests, and to make preliminary predictions on the current level of immunity and inflammation in the body. The association of HGS with the SII in this study has been preliminarily confirmed, and future studies should incorporate strength testing of different muscle groups and assessment of skeletal muscle mass to further explore the relationships among muscle strength, immunity, and inflammation.

## Figures and Tables

**Table 1:** Demographic characteristics stratified by Quartile of SII (N = 8232).

Characteristic	Overall	Quartile 1	Quartile 2	Quartile 3	Quartile 4	*p*-Value
N	8232	2058	2058	2058	2058	
**HGS, mean (SE)**	2.64 (0.02)	2.80 (0.03)	2.71 (0.02)	2.64 (0.03)	2.43 (0.03)	<0.0001
**SII, mean (SE)**	528.93 (7.29)	243.93 (1.48)	382.67 (0.81)	529.65 (1.16)	915.28 (8.39)	<0.0001
**Sex(%)**						<0.0001
Female	4100	896 (44.72)	1011 (49.49)	1061 (49.29)	1132 (56.04)	
Male	4132	1162 (55.28)	1047 (50.51)	997 (50.71)	926 (43.96)	
**Age (%)**						0.02
<20	250	59 (1.70)	75 (2.18)	56 (1.47)	60 (1.89)	
20–29	1409	380 (21.31)	357 (18.21)	351 (18.67)	321 (15.85)	
30–39	1388	345 (17.65)	365 (18.16)	356 (15.26)	322 (15.00)	
40–49	1350	299 (16.03)	345 (17.41)	363 (20.38)	343 (18.79)	
50–59	1320	341 (18.99)	316 (18.24)	336 (20.11)	327 (18.42)	
≥60	2515	634 (24.32)	600 (25.80)	596 (24.11)	685 (30.04)	
**Race (%)**						<0.0001
mexican	921	203 (7.89)	223 (7.25)	258 (8.04)	237 (7.62)	
white	3563	675 (61.38)	885 (69.97)	965 (71.46)	1038 (73.34)	
black	1825	723 (18.42)	425 (9.45)	347 (7.71)	330 (7.17)	
other	1923	457 (12.31)	525 (13.33)	488 (12.78)	453 (11.88)	
**BMI (%)**						<0.0001
<25	2560	705 (33.75)	674 (32.28)	609 (27.43)	572 (27.41)	
25–29.9	2627	679 (34.18)	690 (35.25)	651 (33.88)	607 (29.42)	
≥30	3045	674 (32.07)	694 (32.47)	798 (38.69)	879 (43.17)	
**Edu (%)**						0.03
Below	1661	439 (15.94)	418 (13.47)	390 (13.00)	414 (14.86)	
High school	1838	483 (22.39)	433 (18.87)	451 (21.29)	471 (21.97)	
Above	4733	1136 (61.67)	1207 (67.67)	1217 (65.71)	1173 (63.17)	
**PIR (%)**						0.92
<1.3	2765	673 (22.45)	696 (23.21)	684 (22.33)	712 (23.64)	
1.3–3.49	2858	733 (36.13)	710 (34.45)	719 (34.49)	696 (34.72)	
≥3.5	2609	652 (41.42)	652 (42.35)	655 (43.17)	650 (41.64)	
**Smoke status (%)**						0.03
former	1891	459 (23.03)	453 (22.98)	470 (24.42)	509 (25.89)	
never	4681	1199 (58.40)	1213 (58.41)	1185 (57.11)	1084 (51.93)	
current	1660	400 (18.57)	392 (18.61)	403 (18.47)	465 (22.18)	
**Alcohol status (%)**						0.03
former	1348	343 (13.27)	314 (11.61)	309 (13.57)	382 (16.49)	
never	1218	339 (12.66)	306 (11.09)	283 (10.15)	290 (11.21)	
mild	2765	691 (35.56)	714 (39.59)	683 (35.29)	677 (33.92)	
moderate	1277	291 (17.42)	328 (17.25)	352 (19.44)	306 (16.78)	
heavy	1624	394 (21.10)	396 (20.46)	431 (21.55)	403 (21.60)	
**Diabetes (%)**						< 0.001
no	6783	1721 (89.03)	1720 (87.40)	1720 (86.36)	1622 (83.02)	
yes	1449	337 (10.97)	338 (12.60)	338 (13.64)	436 (16.98)	
**Hypertension (%)**						< 0.001
no	4837	1236 (65.97)	1280 (64.82)	1223 (61.64)	1098 (55.55)	
yes	3395	822 (34.03)	778 (35.18)	835 (38.36)	960 (44.45)	
**Hyperlipidemia (%)**						0.03
no	2575	720 (33.58)	649 (31.43)	629 (29.86)	577 (28.13)	
yes	5657	1338 (66.42)	1409 (68.57)	1429 (70.14)	1481 (71.87)	

Continuous variables are expressed as weighted means (standard error), and categorical variables are expressed as weighted population (percentage), unless otherwise indicated. Differences between groups by sex were compared by *t*-test and chi-square test. HGS, handgrip strength; SII, systemic immune-inflammatory index; PIR, poverty-to-income ratio; Edu, educational level; BMI, body mass index; Systemic immune-inflammatory index quartile ranges: quartile 1: 1.53–317.74; quartile 2: 317.75–445.51; quartile 3: 445.52–632.56; quartile 4: 632.57–8464.

**Table 2:** Weighted generalized linear regression analysis of the relationship between grip strength and SII.

	Model I	Model II	Model III
	β (95%CI) *p*-Value	β (95%CI) *p*-Value	β (95%CI) *p*-Value
Grip Strength (Quartile)			
Quartile 1	Ref	Ref	Ref
Quartile 2	−49.36 (−71.89, −26.84) <0.0010	−38.96 (−65.17, −12.76) 0.0100	−35.75 (−66.93, −4.56) 0.0300
Quartile 3	−94.28 (−120.53, −68.03) <0.0001	−83.86 (−114.97, −52.76) <0.0001	−79.49 (−115.44, −43.55) <0.0010
Quartile 4	−103.61 (−126.53, −80.68) <0.0001	−85.88 (−130.09, −41.67) <0.0010	−77.31 (−129.58, −25.04) 0.0100
*p* for trend	<0.0001	<0.0010	<0.0100
Stratified by sex			
Male			
Quartile 1	Ref	Ref	Ref
Quartile 2	−75.83 (−112.64, −39.02) <0.0010	−61.09 (−95.12, −27.06) <0.0100	−61.03 (−101.14, −20.92) 0.0100
Quartile 3	−81.01 (−121.86, −40.16) <0.0010	−64.36 (−112.23, −16.49) 0.0100	−61.28 (−116.71, −5.86) 0.0400
Quartile 4	−96.36 (−137.41, −55.31) <0.0001	−65.42 (−111.47, −19.36) 0.0100	−64.36 (−118.25, −10.46) 0.0300
*p* for trend	<0.0010	0.0200	0.0400
Female			
Quartile 1	Ref	Ref	Ref
Quartile 2	−39.41 (−76.26, −2.56) 0.0400	−33.2 (−77.23, 10.82) 0.1300	−24.91 (−73.03, 23.20) 0.2500
Quartile 3	−86.3 (−119.82, −52.79) <0.0001	−72.68 (−121.73, −23.63) 0.0100	−62.01 (−117.07, −6.94) 0.0300
Quartile 4	−95.1 (−131.09, −59.11) <0.0001	−86.56 (−143.18, −29.95) 0.0100	−74.94 (−137.21, −12.66) 0.0300
*p* for trend	<0.0001	<0.0100	0.0100

Model I: No covariates were adjusted; Model II: Adjusted for sex, age, race, BMI, education, and PIR; Model III: Adjusted for sex, age, race, BMI, education, PIR, smoking, alcohol, diabetes, hypertension, and hyperlipidemia. Handgrip strength quartile ranges: quartile 1: 0.41–1.87; quartile 2: 1.88–2.48; quartile 3: 2.49–3.20; quartile 4: 3.21–7.02.

## Data Availability

Publicly available datasets were analyzed in this study. This data can be found here: https://wwwn.cdc.gov/nchs/nhanes/ (accessed on 1 May 2022).
